# Benzyl *N*-{2-[5-(4-chloro­phen­yl)-1,2,4-oxadiazol-3-yl]propan-2-yl}carbamate

**DOI:** 10.1107/S1600536811001504

**Published:** 2011-01-15

**Authors:** Hoong-Kun Fun, V. Sumangala, G. K. Nagaraja, Boja Poojary, Suchada Chantrapromma

**Affiliations:** a X-ray Crystallography Unit, School of Physics, Universiti Sains Malaysia, 11800 USM, Penang, Malaysia; b Department of Chemistry, Mangalore University, Mangalagangotri 574 199, Karnatak State, India; c Crystal Materials Research Unit, Department of Chemistry, Faculty of Science, Prince of Songkla University, Hat-Yai, Songkhla 90112, Thailand

## Abstract

In the title 1,2,4-oxadiazole derivative, C_19_H_18_ClN_3_O_3_, the 1,2,4-oxadiazole ring makes dihedral angles of 12.83 (8) and 4.89 (8)°, respectively, with the benzyl and 4-chloro­phenyl rings, while the dihedral angle between the benzyl and 4-chloro­phenyl rings is 11.53 (7)°. In the crystal, mol­ecules are linked by N—H⋯N hydrogen bonds into helical chains along the *b* axis. A weak C—H⋯π inter­action is also present.

## Related literature

For bond-length data, see: Allen *et al.* (1987 ▶). For background to and applications of 1,2,4-oxadiazole derivatives, see: Chen *et al.* (1994 ▶); Chimirri *et al.* (1996 ▶); Clitherow *et al.* (1996 ▶); Nicolaides *et al.* (1998 ▶); Saunders *et al.* (1990 ▶); Showell *et al.* (1991 ▶); Swain *et al.* (1991 ▶); Tully *et al.* (1991 ▶); Watjen *et al.* (1989 ▶). For a related structure, see: Fun *et al.* (2011 ▶).
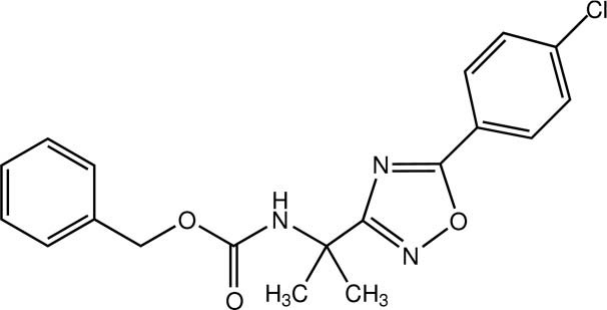


## Experimental

### 

#### Crystal data

C_19_H_18_ClN_3_O_3_
*M* *_r_* = 371.81
Orthorhombic, 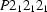

*a* = 7.7501 (1) Å
*b* = 11.0052 (2) Å
*c* = 20.9834 (3) Å
*V* = 1789.70 (5) Å^3^
*Z* = 4
Mo *K*α radiation
μ = 0.24 mm^−1^
*T* = 297 K
0.41 × 0.35 × 0.21 mm

#### Data collection

Bruker APEXII CCD area-detector diffractometer
Absorption correction: multi-scan (*SADABS*; Bruker, 2005 ▶) *T*_min_ = 0.908, *T*_max_ = 0.951
15995 measured reflections
4556 independent reflections
3620 reflections with *I* > 2σ(*I*)
*R*_int_ = 0.028

#### Refinement

*R*[*F* ^2^ > 2σ(*F* ^2^)] = 0.036
*wR*(*F* ^2^) = 0.087
*S* = 1.02
4556 reflections
241 parameters
H atoms treated by a mixture of independent and constrained refinement
Δρ_max_ = 0.14 e Å^−3^
Δρ_min_ = −0.21 e Å^−3^
Absolute structure: Flack (1983 ▶), 1950 Friedel pairs
Flack parameter: 0.01 (6)

### 

Data collection: *APEX2* (Bruker, 2005 ▶); cell refinement: *SAINT* (Bruker, 2005 ▶); data reduction: *SAINT*; program(s) used to solve structure: *SHELXTL* (Sheldrick, 2008 ▶); program(s) used to refine structure: *SHELXTL*; molecular graphics: *SHELXTL*; software used to prepare material for publication: *SHELXTL* and *PLATON* (Spek, 2009 ▶).

## Supplementary Material

Crystal structure: contains datablocks global, I. DOI: 10.1107/S1600536811001504/is2659sup1.cif

Structure factors: contains datablocks I. DOI: 10.1107/S1600536811001504/is2659Isup2.hkl

Additional supplementary materials:  crystallographic information; 3D view; checkCIF report

## Figures and Tables

**Table 1:** Hydrogen-bond geometry (Å, °) *Cg*1 is the centroid of the C12–C17 ring.

*D*—H⋯*A*	*D*—H	H⋯*A*	*D*⋯*A*	*D*—H⋯*A*
N3—H1*N*3⋯N2^i^	0.840 (16)	2.428 (15)	3.2492 (18)	165.7 (14)
C16—H16*A*⋯*Cg*1^ii^	0.93	2.91	3.6700 (18)	140

Symmetry codes: (i) 
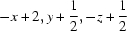
; (ii) 
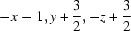
.

**Fig. 1.**
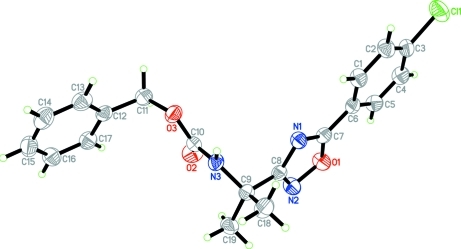
The molecular structure of the title compound, showing 40% probability displacement ellipsoids and the atom-numbering scheme.

**Fig. 2.**
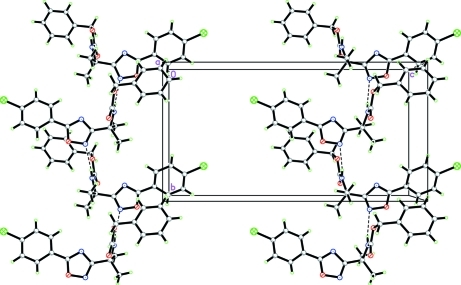
The crystal packing of the title compound viewed along the a axis, showing helical chains along the b axis. N—H···N hydrogen bonds are shown as dashed lines.

## supplementary crystallographic information

### Comment

The 1,2,4-oxadiazole ring occurs frequently in biologically active synthetic compounds and 1,2,4-oxadiazole derivatives are suggested as potential agonists for cortical muscarinic (Saunders *et al.*, 1990; Showell *et al.*, 1991), benzodiazepine (Watjen *et al.*, 1989; Tully *et al.*, 1991) and 5-HT1D (5-hydroxytryptamine) as receptors (Chen *et al.*, 1994) as well as antagonists for 5-HT3 (Swain *et al.*, 1991) or histamine H3 receptors (Clitherow *et al.*, 1996). They also demonstrate anti-inflammatory (Nicolaides *et al.*, 1998) and antitumor activities (Chimirri *et al.*, 1996). These interesting and important properties of 1,2,4-oxadiazole derivatives lead us to synthesize the title compound (I) and its crystal structure was reported.

In the molecule of (I), (Fig. 1), the 1,2,4-oxadiazole ring is nearly co-planar with the 4-chlorophenyl with a dihedral angle between the two rings being 4.89 (8)°, whereas it is inclined to the benzyl (C11–C17) unit with a dihedral angle of 12.48 (9)°. The dihedral angle between the benzyl group and the phenyl (C1–C6) ring is 11.53 (7)°. The oxycarbonylamino unit (atoms C10, N3, O2 and O3) is planar with an *r.m.s*. 0.0009 (1) Å. This unit makes dihedral angles of 79.99 (9), 89.15 (9) and 89.64 (9)°, respectively, with the benzyl, 1,2,4-oxadiazole and 4-chlorophenyl rings. The bond distances are of normal values (Allen *et al.*, 1987) and are comparable to the related structure (Fun *et al.*, 2011).

In the crystal packing (Fig. 2), the molecules are linked by by N—H···N hydrogen bonds (Table 1) into helical chains along the *b* axis. The crystal is stabilized by N—H···O hydrogen bonds and weak C—H···π interactions (Table 1).

### Experimental

The title compound was synthesized by taking benzyl [(2*E*)-2-amino-2-(hydroxyimino)-1,1-dimethylethyl]carbamate (0.1 mole) in 5 mol volume of pyridine and treated with 0.1 mole of 4-chlorobenzoylchloride. The reaction mixture is heated to reflux for 3–4 hrs. After the reaction is completed, the excess pyridine is distilled out under low pressure and the resultant quenched onto ice water. The solid thus formed is filtered and washed with water. The sample is dried and recrystallized in ethanol yielding 95% product. Colorless plate-shaped single crystals of the title compound suitable for *x*-ray structure determination were recrystalized from ethanol by the slow evaporation of the solvent at room temperature after several days (m.p. 405–409 K).

### Refinement

NH hydrogen atom is located in a difference map and refined isotropically. The remaining H atoms were positioned geometrically and allowed to ride on their parent atoms, with *d*(C—H) = 0.93 Å for aromatic and 0.96 Å for CH_3_ atoms. The *U*_iso_ values were constrained to be 1.5*U*_eq_ of the carrier atom for methyl H atoms and 1.2*U*_eq_ for the remaining H atoms. A rotating group model was used for the methyl groups. A total of 1950 Friedel pairs were used to determined the absolute configuration

### Crystal data

**Table:**

C_19_H_18_ClN_3_O_3_	*D*_x_ = 1.380 Mg m^−^^3^
*M**_r_* = 371.81	Melting point = 405–409 K
Orthorhombic, *P*2_1_2_1_2_1_	Mo *K*α radiation, λ = 0.71073 Å
Hall symbol: P 2ac 2ab	Cell parameters from 4556 reflections
*a* = 7.7501 (1) Å	θ = 1.9–28.5°
*b* = 11.0052 (2) Å	µ = 0.24 mm^−^^1^
*c* = 20.9834 (3) Å	*T* = 297 K
*V* = 1789.70 (5) Å^3^	Plate, colorless
*Z* = 4	0.41 × 0.35 × 0.21 mm
*F*(000) = 776	

### Data collection

**Table:**

Bruker APEXII CCD area-detector diffractometer	4556 independent reflections
Radiation source: sealed tube	3620 reflections with *I* > 2σ(*I*)
graphite	*R*_int_ = 0.028
φ and ω scans	θ_max_ = 28.5°, θ_min_ = 1.9°
Absorption correction: multi-scan (*SADABS*; Bruker, 2005)	*h* = −10→8
*T*_min_ = 0.908, *T*_max_ = 0.951	*k* = −14→14
15995 measured reflections	*l* = −23→28

### Refinement

**Table:**

Refinement on *F*^2^	Secondary atom site location: difference Fourier map
Least-squares matrix: full	Hydrogen site location: inferred from neighbouring sites
*R*[*F*^2^ > 2σ(*F*^2^)] = 0.036	H atoms treated by a mixture of independent and constrained refinement
*wR*(*F*^2^) = 0.087	*w* = 1/[σ^2^(*F*_o_^2^) + (0.0458*P*)^2^ + 0.0092*P*] where *P* = (*F*_o_^2^ + 2*F*_c_^2^)/3
*S* = 1.02	(Δ/σ)_max_ = 0.001
4556 reflections	Δρ_max_ = 0.14 e Å^−^^3^
241 parameters	Δρ_min_ = −0.21 e Å^−^^3^
0 restraints	Absolute structure: Flack (1983), 1950 Friedel pairs
Primary atom site location: structure-invariant direct methods	Flack parameter: 0.01 (6)

### Special details

**Table:**

Geometry. All e.s.d.'s (except the e.s.d. in the dihedral angle between two l.s. planes) are estimated using the full covariance matrix. The cell e.s.d.'s are taken into account individually in the estimation of e.s.d.'s in distances, angles and torsion angles; correlations between e.s.d.'s in cell parameters are only used when they are defined by crystal symmetry. An approximate (isotropic) treatment of cell e.s.d.'s is used for estimating e.s.d.'s involving l.s. planes.
Refinement. Refinement of *F*^2^ against ALL reflections. The weighted *R*-factor *wR* and goodness of fit *S* are based on *F*^2^, conventional *R*-factors *R* are based on *F*, with *F* set to zero for negative *F*^2^. The threshold expression of *F*^2^ > σ(*F*^2^) is used only for calculating *R*-factors(gt) *etc*. and is not relevant to the choice of reflections for refinement. *R*-factors based on *F*^2^ are statistically about twice as large as those based on *F*, and *R*- factors based on ALL data will be even larger.

### Fractional atomic coordinates and isotropic or equivalent isotropic displacement parameters (Å^2^)

**Table:**

	*x*	*y*	*z*	*U*_iso_*/*U*_eq_	
Cl1	1.06337 (9)	0.74415 (5)	−0.14993 (2)	0.08010 (19)	
O1	0.90644 (16)	0.42996 (9)	0.11883 (5)	0.0490 (3)	
O2	0.62272 (16)	0.60054 (11)	0.29142 (7)	0.0631 (4)	
O3	0.71211 (14)	0.79437 (9)	0.30763 (6)	0.0501 (3)	
N1	1.00418 (18)	0.59694 (10)	0.16396 (6)	0.0422 (3)	
N2	0.89957 (19)	0.41084 (11)	0.18577 (6)	0.0487 (3)	
N3	0.90781 (17)	0.65050 (11)	0.29774 (6)	0.0401 (3)	
C1	1.0718 (2)	0.70094 (14)	0.03799 (7)	0.0470 (4)	
H1A	1.1094	0.7446	0.0733	0.056*	
C2	1.0945 (2)	0.74801 (15)	−0.02232 (8)	0.0522 (4)	
H2A	1.1470	0.8233	−0.0279	0.063*	
C3	1.0385 (2)	0.68209 (16)	−0.07420 (7)	0.0499 (4)	
C4	0.9627 (2)	0.56952 (14)	−0.06747 (8)	0.0517 (4)	
H4A	0.9283	0.5255	−0.1031	0.062*	
C5	0.9386 (2)	0.52308 (13)	−0.00694 (7)	0.0469 (4)	
H5A	0.8859	0.4478	−0.0017	0.056*	
C6	0.9930 (2)	0.58868 (12)	0.04632 (7)	0.0393 (3)	
C7	0.96954 (19)	0.54258 (12)	0.11114 (7)	0.0386 (3)	
C8	0.9582 (2)	0.51195 (12)	0.20882 (7)	0.0371 (3)	
C9	0.9872 (2)	0.53376 (12)	0.27932 (7)	0.0417 (4)	
C10	0.7365 (2)	0.67374 (13)	0.29808 (7)	0.0414 (3)	
C11	0.5372 (2)	0.83404 (16)	0.32187 (8)	0.0528 (4)	
H11A	0.4552	0.7744	0.3064	0.063*	
H11B	0.5141	0.9106	0.3007	0.063*	
C12	0.51699 (19)	0.84918 (13)	0.39282 (7)	0.0423 (3)	
C13	0.5898 (2)	0.94799 (14)	0.42406 (9)	0.0545 (4)	
H13A	0.6480	1.0070	0.4008	0.065*	
C14	0.5766 (3)	0.95920 (16)	0.48927 (9)	0.0621 (5)	
H14A	0.6270	1.0253	0.5096	0.074*	
C15	0.4903 (3)	0.87436 (17)	0.52416 (9)	0.0591 (5)	
H15A	0.4826	0.8825	0.5682	0.071*	
C16	0.4146 (2)	0.77656 (16)	0.49429 (8)	0.0572 (4)	
H16A	0.3541	0.7191	0.5179	0.069*	
C17	0.4292 (2)	0.76457 (13)	0.42877 (8)	0.0496 (4)	
H17A	0.3788	0.6981	0.4087	0.060*	
C18	1.1805 (2)	0.54871 (16)	0.28978 (9)	0.0562 (5)	
H18A	1.2220	0.6155	0.2647	0.084*	
H18B	1.2389	0.4756	0.2773	0.084*	
H18C	1.2024	0.5645	0.3340	0.084*	
C19	0.9170 (3)	0.42786 (14)	0.31875 (8)	0.0592 (5)	
H19A	0.7970	0.4162	0.3092	0.089*	
H19B	0.9302	0.4456	0.3633	0.089*	
H19C	0.9799	0.3552	0.3085	0.089*	
H1N3	0.973 (2)	0.7115 (14)	0.2985 (7)	0.036 (4)*	

### Atomic displacement parameters (Å^2^)

**Table:**

	*U*^11^	*U*^22^	*U*^33^	*U*^12^	*U*^13^	*U*^23^
Cl1	0.1106 (5)	0.0857 (4)	0.0440 (2)	0.0012 (3)	0.0132 (3)	0.0075 (2)
O1	0.0635 (7)	0.0416 (5)	0.0418 (6)	−0.0124 (5)	−0.0050 (6)	−0.0044 (4)
O2	0.0508 (7)	0.0552 (7)	0.0833 (10)	−0.0086 (6)	−0.0013 (7)	0.0010 (6)
O3	0.0486 (7)	0.0433 (5)	0.0584 (7)	0.0076 (5)	0.0118 (6)	0.0012 (5)
N1	0.0508 (8)	0.0373 (6)	0.0384 (7)	−0.0039 (5)	0.0004 (6)	−0.0046 (5)
N2	0.0609 (9)	0.0431 (7)	0.0422 (7)	−0.0097 (6)	−0.0013 (7)	−0.0010 (5)
N3	0.0438 (8)	0.0338 (6)	0.0425 (7)	−0.0013 (6)	0.0046 (6)	−0.0040 (5)
C1	0.0536 (10)	0.0451 (8)	0.0422 (8)	−0.0064 (7)	−0.0001 (8)	−0.0097 (6)
C2	0.0575 (10)	0.0478 (8)	0.0514 (9)	−0.0079 (8)	0.0081 (8)	−0.0012 (7)
C3	0.0530 (10)	0.0577 (9)	0.0389 (8)	0.0070 (8)	0.0062 (8)	−0.0006 (7)
C4	0.0598 (11)	0.0534 (9)	0.0420 (8)	0.0038 (8)	−0.0090 (8)	−0.0110 (7)
C5	0.0536 (11)	0.0415 (8)	0.0457 (8)	−0.0013 (7)	−0.0070 (8)	−0.0065 (6)
C6	0.0399 (8)	0.0388 (7)	0.0390 (8)	0.0034 (6)	0.0000 (7)	−0.0060 (5)
C7	0.0376 (8)	0.0352 (7)	0.0431 (8)	−0.0002 (6)	−0.0026 (7)	−0.0057 (6)
C8	0.0374 (8)	0.0328 (6)	0.0410 (7)	0.0000 (6)	0.0007 (6)	−0.0021 (5)
C9	0.0507 (10)	0.0357 (7)	0.0389 (8)	0.0043 (6)	0.0017 (7)	−0.0032 (6)
C10	0.0496 (9)	0.0419 (7)	0.0327 (7)	0.0033 (7)	0.0047 (7)	0.0044 (6)
C11	0.0474 (10)	0.0591 (10)	0.0518 (10)	0.0146 (8)	0.0031 (8)	0.0053 (7)
C12	0.0345 (8)	0.0425 (7)	0.0499 (9)	0.0088 (6)	0.0019 (7)	0.0026 (6)
C13	0.0503 (10)	0.0438 (8)	0.0692 (12)	−0.0025 (8)	0.0048 (9)	0.0023 (8)
C14	0.0618 (12)	0.0539 (10)	0.0705 (12)	0.0008 (9)	−0.0075 (11)	−0.0185 (8)
C15	0.0585 (12)	0.0680 (11)	0.0507 (10)	0.0107 (9)	0.0034 (9)	−0.0074 (8)
C16	0.0589 (11)	0.0567 (10)	0.0561 (10)	−0.0002 (9)	0.0101 (9)	0.0080 (7)
C17	0.0506 (10)	0.0423 (8)	0.0560 (9)	−0.0043 (8)	0.0001 (8)	−0.0035 (7)
C18	0.0540 (11)	0.0618 (10)	0.0528 (10)	0.0117 (8)	−0.0128 (9)	−0.0138 (8)
C19	0.0924 (15)	0.0401 (8)	0.0452 (9)	0.0039 (9)	0.0064 (10)	0.0028 (6)

### Geometric parameters (Å, °)

**Table:**

Cl1—C3	1.7404 (16)	C8—C9	1.515 (2)
O1—C7	1.3422 (16)	C9—C18	1.523 (2)
O1—N2	1.4213 (17)	C9—C19	1.529 (2)
O2—C10	1.2023 (19)	C11—C12	1.506 (2)
O3—C10	1.3558 (18)	C11—H11A	0.9700
O3—C11	1.455 (2)	C11—H11B	0.9700
N1—C7	1.2877 (18)	C12—C17	1.378 (2)
N1—C8	1.3740 (19)	C12—C13	1.390 (2)
N2—C8	1.2955 (18)	C13—C14	1.378 (3)
N3—C10	1.352 (2)	C13—H13A	0.9300
N3—C9	1.4758 (18)	C14—C15	1.362 (3)
N3—H1N3	0.841 (16)	C14—H14A	0.9300
C1—C2	1.379 (2)	C15—C16	1.377 (3)
C1—C6	1.389 (2)	C15—H15A	0.9300
C1—H1A	0.9300	C16—C17	1.386 (2)
C2—C3	1.378 (2)	C16—H16A	0.9300
C2—H2A	0.9300	C17—H17A	0.9300
C3—C4	1.378 (2)	C18—H18A	0.9600
C4—C5	1.382 (2)	C18—H18B	0.9600
C4—H4A	0.9300	C18—H18C	0.9600
C5—C6	1.3957 (19)	C19—H19A	0.9600
C5—H5A	0.9300	C19—H19B	0.9600
C6—C7	1.463 (2)	C19—H19C	0.9600
			
C7—O1—N2	105.61 (10)	O2—C10—O3	124.84 (16)
C10—O3—C11	116.99 (13)	N3—C10—O3	108.82 (14)
C7—N1—C8	102.66 (12)	O3—C11—C12	109.46 (13)
C8—N2—O1	103.21 (11)	O3—C11—H11A	109.8
C10—N3—C9	125.11 (13)	C12—C11—H11A	109.8
C10—N3—H1N3	116.2 (10)	O3—C11—H11B	109.8
C9—N3—H1N3	116.6 (10)	C12—C11—H11B	109.8
C2—C1—C6	120.39 (14)	H11A—C11—H11B	108.2
C2—C1—H1A	119.8	C17—C12—C13	118.11 (15)
C6—C1—H1A	119.8	C17—C12—C11	121.18 (14)
C3—C2—C1	119.14 (15)	C13—C12—C11	120.70 (15)
C3—C2—H2A	120.4	C14—C13—C12	120.56 (16)
C1—C2—H2A	120.4	C14—C13—H13A	119.7
C2—C3—C4	121.76 (15)	C12—C13—H13A	119.7
C2—C3—Cl1	118.67 (13)	C15—C14—C13	120.61 (17)
C4—C3—Cl1	119.56 (13)	C15—C14—H14A	119.7
C3—C4—C5	118.97 (14)	C13—C14—H14A	119.7
C3—C4—H4A	120.5	C14—C15—C16	120.02 (18)
C5—C4—H4A	120.5	C14—C15—H15A	120.0
C4—C5—C6	120.26 (15)	C16—C15—H15A	120.0
C4—C5—H5A	119.9	C15—C16—C17	119.42 (16)
C6—C5—H5A	119.9	C15—C16—H16A	120.3
C1—C6—C5	119.47 (14)	C17—C16—H16A	120.3
C1—C6—C7	118.68 (13)	C12—C17—C16	121.27 (15)
C5—C6—C7	121.84 (14)	C12—C17—H17A	119.4
N1—C7—O1	113.68 (13)	C16—C17—H17A	119.4
N1—C7—C6	127.82 (13)	C9—C18—H18A	109.5
O1—C7—C6	118.50 (12)	C9—C18—H18B	109.5
N2—C8—N1	114.83 (13)	H18A—C18—H18B	109.5
N2—C8—C9	123.53 (13)	C9—C18—H18C	109.5
N1—C8—C9	121.50 (12)	H18A—C18—H18C	109.5
N3—C9—C8	109.39 (12)	H18B—C18—H18C	109.5
N3—C9—C18	106.14 (13)	C9—C19—H19A	109.5
C8—C9—C18	107.66 (13)	C9—C19—H19B	109.5
N3—C9—C19	111.94 (13)	H19A—C19—H19B	109.5
C8—C9—C19	110.79 (12)	C9—C19—H19C	109.5
C18—C9—C19	110.72 (15)	H19A—C19—H19C	109.5
O2—C10—N3	126.34 (14)	H19B—C19—H19C	109.5
			
C7—O1—N2—C8	−0.08 (16)	C10—N3—C9—C18	−177.07 (15)
C6—C1—C2—C3	−0.2 (3)	C10—N3—C9—C19	−56.2 (2)
C1—C2—C3—C4	−1.0 (3)	N2—C8—C9—N3	−131.00 (16)
C1—C2—C3—Cl1	178.49 (14)	N1—C8—C9—N3	53.48 (19)
C2—C3—C4—C5	1.6 (3)	N2—C8—C9—C18	114.08 (17)
Cl1—C3—C4—C5	−177.88 (13)	N1—C8—C9—C18	−61.44 (18)
C3—C4—C5—C6	−1.0 (3)	N2—C8—C9—C19	−7.1 (2)
C2—C1—C6—C5	0.7 (2)	N1—C8—C9—C19	177.35 (15)
C2—C1—C6—C7	−179.32 (15)	C9—N3—C10—O2	9.9 (3)
C4—C5—C6—C1	−0.1 (2)	C9—N3—C10—O3	−170.43 (13)
C4—C5—C6—C7	179.93 (15)	C11—O3—C10—O2	11.3 (2)
C8—N1—C7—O1	−0.38 (18)	C11—O3—C10—N3	−168.37 (12)
C8—N1—C7—C6	−179.60 (15)	C10—O3—C11—C12	98.05 (16)
N2—O1—C7—N1	0.30 (18)	O3—C11—C12—C17	−104.15 (17)
N2—O1—C7—C6	179.60 (13)	O3—C11—C12—C13	74.49 (18)
C1—C6—C7—N1	4.6 (2)	C17—C12—C13—C14	1.1 (2)
C5—C6—C7—N1	−175.44 (16)	C11—C12—C13—C14	−177.57 (16)
C1—C6—C7—O1	−174.60 (14)	C12—C13—C14—C15	−0.7 (3)
C5—C6—C7—O1	5.4 (2)	C13—C14—C15—C16	−0.3 (3)
O1—N2—C8—N1	−0.16 (18)	C14—C15—C16—C17	0.9 (3)
O1—N2—C8—C9	−175.95 (14)	C13—C12—C17—C16	−0.5 (2)
C7—N1—C8—N2	0.33 (19)	C11—C12—C17—C16	178.18 (15)
C7—N1—C8—C9	176.22 (14)	C15—C16—C17—C12	−0.5 (3)
C10—N3—C9—C8	67.03 (18)		

### Hydrogen-bond geometry (Å, °)

**Table:**

*Cg*1 is the centroid of the C12–C17 ring.

**Table:**

*D*—H···*A*	*D*—H	H···*A*	*D*···*A*	*D*—H···*A*
N3—H1N3···N2^i^	0.840 (16)	2.428 (15)	3.2492 (18)	165.7 (14)
C16—H16A···Cg1^ii^	0.93	2.91	3.6700 (18)	140

Symmetry codes: (i) −*x*+2, *y*+1/2, −*z*+1/2; (ii) −*x*−1, *y*+3/2, −*z*+3/2.
